# The Role of Nucleotides and Purinergic Signaling in Apoptotic Cell Clearance – Implications for Chronic Inflammatory Diseases

**DOI:** 10.3389/fimmu.2014.00656

**Published:** 2014-12-23

**Authors:** Jin Chen, Yi Zhao, Yi Liu

**Affiliations:** ^1^Department of Rheumatology and Immunology, West China Hospital, Sichuan University, Chengdu, Sichuan, China

**Keywords:** ATP, extracellular nucleotides, purinergic signaling, apoptotic cell clearance, chronic inflammation

## Abstract

Billions of cells undergo apoptosis every day in healthy individuals. A prompt removal of dying cells prevents the release of pro-inflammatory intracellular content and progress to secondary necrosis. Thus, inappropriate clearance of apoptotic cells provokes autoimmunity and has been associated with many chronic inflammatory diseases. Recent studies have suggested that extracellular adenosine 5′-triphosphate and related nucleotides play an important role in the apoptotic clearance process. Here, we review the current understanding of nucleotides and purinergic receptors in apoptotic cell clearance and the potential therapeutic targets of purinergic receptor subtypes in inflammatory conditions.

## Introduction

Apoptosis occurs in all multicellular organisms and plays a role in getting rid of superfluous and senescent cells during the development of an organism, tissue homeostasis, and pathogenic processes (1). In contrast to necrosis, apoptosis is a highly organized and fine-tuned process, and is, therefore, usually referred to as programed cell death. Besides the physiological process, apoptotic cells are also observed in tumors (2), atherosclerotic plaques (3, 4), and autoimmune diseases (5, 6). Under normal conditions, the apoptotic cell removal is performed very efficiently and fast by neighboring or recruited phagocytes and is important for maintaining the function of tissues (6, 7). Dying cells can undergo secondary necrosis if not cleared promptly and the release of intracellular contents has been linked to many human inflammatory diseases (8, 9). Moreover, apoptotic cells have been shown to have anti-inflammatory and regenerative effects (10).

Damaged tissues and dying cells can release nucleotides, which are increasingly viewed as a new class of regulators of the immune system. The class of purinergic receptors is involved in a wide range of phagocytic and chemotactic processes (11). Moreover, the purinergic signaling is an important regulatory mechanism in several inflammatory diseases (12). Several studies provide strong evidence that nucleotides and activated purinergic receptors are linked to the pathogenesis of many chronic inflammatory diseases. This review will discuss the apoptotic cell clearance with special emphasis the specific role of nucleotides and the purinergic receptors in the development of chronic inflammatory diseases related with abnormal clearance of apoptotic cells.

## Components of Purinergic Signaling

### Extracellular ATP release and metabolism

Damaged tissues and dying cells can release adenosine 5′-triphosphate (ATP) as a danger signal that triggers a variety of inflammatory responses. Moreover, ATP can also actively be released from intact cells in response to mechanical deformation, hypoxia or acetylcholine, which do not damage the cell (7, 13, 14). For example, ATP release from intact cells was firstly reported for neuronal cells, which release ATP into the cleft of chemical synapses (15). However, the underlying mechanism has been shown to be very complex and includes stretch-activated channels, voltage-dependent anion channels, P2X7 receptors, and connexin and pannexin hemichannels (16).

Contrasting to intracellular ATP, primarily utilized as energy, extracellular ATP is considered to be a powerful signaling molecule through the nucleotide-selective P2 receptors. Extracellular ATP is rapidly metabolized to adenosine by ectonucleotidases (17). The ectonucleotidases consist of four family types including (i) ectonucleotide pyrophosphatase/phosphodiesterase (E-NPP) family, (ii) ectonucleoside triphosphate diphosphohydrolase (E-NTPDase) family, (iii) alkaline phosphatases (AP), and (iv) ecto-5′-nucleotidase (also known as CD73) (17, 18). Extracellular adenosine, an intermediate metabolite of nucleotides, can undergo three processes: (i) conversion to inosine by adenosine deaminase, (ii) reconversion to AMP by adenosine kinase, and (iii) cellular reuptake through concentrative nucleoside transporters (CNTs) or equilibrative nucleoside transporters (ENTs) (17, 19, 20).

### Purinergic receptors

Purinergic receptors have been widely studied in signaling systems in response to extracellular ATP and related nucleotides. Purinergic receptors consist of three major families based on their structural and biological properties (21). The G-protein-coupled P2Y receptors (P2YRs) recognize ATP and several other nucleotides, including ADP, UTP, UDP, and UDP-glucose (22). P2X receptors (P2XRs) function as ATP-gated ion channels that facilitate the influx and efflux of extracellular cations, including calcium ions, which only respond to ATP (22, 23). To date, P2YRs consist of eight subtypes, a family of P2Y1, P2Y2, P2Y4, P2Y6, P2Y11, P2Y12, P2Y13, and P2Y14Rs. P2XRs have seven subunits that may form six homomeric (P2X1–P2X5Rs and P2X7R), and at least seven heteromeric P2X1/2, P2X1/4, P2X1/5, P2X2/3, P2X2/5, P2X2/6, and P2X4/6Rs receptors (23–25). The conversion of ATP/ADP to adenosine by ectonucleotidases terminates P2R signaling within the extracellular compartment. Adenosine can signal through four distinct G-protein-coupled receptors (P1 receptors): adenosine A1 receptor (A1), adenosine A2a receptor (A2a), adenosine A2b receptor (A2b), and adenosine A3 receptor (A3) (Table 1) (26–28). The purinergic receptor subtypes are widely distributed throughout the immune cells and the central nervous system (CNS) (Table 1) (29–31).

**Table 1:** **Characteristics of purinergic receptors [Modified from Ref. (15, 29, 32, 33)]**.

Receptor		Distribution	Functions
P2Y	P2Y1	Platelets, immune cells, epithelial and endothelial cells, and osteoclasts	Platelet aggregation, smooth muscle relaxation, and bone resorption
	P2Y2	Astrocytes, immune cells, epithelial and endothelial cells, and osteoblasts	Promotes apoptotic cell removal; mediates airway surfactant secretion and epithelial cell chloride secretion; vasodilatation through endothelium and vasoconstriction through smooth muscle; bone remodeling; role in neutrophil chemotaxis; and chronic inflammation
	P2Y4	Endothelial and epithelial cells	Epithelial chloride transport regulation; vasodilatation through endothelium
	P2Y6	Activated microglia, T cells, and epithelial cells	Enhances microglial phagocytic capacity; modulating cytokines release; epithelium NaCl secretion; epithelial proliferation; and role in colitis
	P2Y11	Dendritic cells, granulocytes	Mediates dendritic cells maturation and migration; granulocytic differentiation
	P2Y12	Platelets and glial cells	Platelet aggregation; dense granule secretion
	P2Y13	Spinal cord microglia, hepatocytes	Regulates lipid metabolism and atherosclerosis
	P2Y14	Hematopoietic cells, immune cells	Hematopoietic stem cells chemotaxis; dendritic cell activation
P2X	P2X1	Platelets, smooth muscle	Platelet activation; smooth muscle contraction
	P2X2	Autonomic and sensory ganglia, retina	Sensory transmission and modulation of synaptic function
	P2X3	Sensory neurons, sympathetic neurons	Mediates sensory transmission; facilitates glutamate release in CNS
	P2X4	Microglial cell, immune cells	Modulates chronic inflammatory and neuropathic pain
	P2X5	Dendritic cells	Mediating cell proliferation and differentiation
	P2X6	Neuron, retina, and myocardial cell	Functions as a heteromeric channel in combination with P2X2 and P2X4 subunits
	P2X7	Immune cells, osteoclasts, and microglia	Mediates apoptosis, cell proliferation and pro-inflammatory cytokine release
P1	A1	Neurons, autonomic nerve terminals	Modulates neurotransmitter release; treatment in cardiac tachycardia
	A2a	B cells, T cells	Anti-inflammatory effect; mediates cytokines release; facilitates neurotransmission; and smooth muscle relaxation
	A2b	Bronchial epithelial cells, cardiomyocytes	Dampens inflammation in allergic and inflammatory disorders; vasodilatation
	A3	Endothelial cells, immune cells, and cardiomyocytes	Mediates anti-inflammatory, anti-ischemic, and antitumor effect

## Apoptotic Cell Recognition and Clearance

Apoptosis is a crucial process during development and regeneration of an organism. The prompt and efficient engulfment of apoptotic cells by phagocytes is necessary to prevent inflammation resulting from uncontrolled release of intracellular contents (34). Apoptotic cell clearance can be subdivided into four general steps: sensing of the apoptotic cell, recognition, engulfment of the corpse, and processing of the engulfed material (7, 35–38). Many key molecules and several molecular pathways have been identified to orchestrate the safe disposal of apoptotic cells. Apoptotic cells release so-called “find me” signals, which are cell-derived chemoattractants to entice phagocytes (9). To date, several proposed “find me” signals released by dying cells have been reported. These include the nucleotides ATP and UTP (39), lysophosphatidylcholine (LPC) (40), fractalkine (CX3CL1) (41), and sphingosine 1-phosphate (S1P) (42). In addition to attracting phagocytes, apoptotic cells are thought to release factors, referred to as “stay away” signals, to exclude inflammatory cells such as neutrophils (43).

At the same time, apoptotic cells also expose phosphatidylserine (PS) on the outer leaflet of the plasma membrane as an “eat-me” signal to promote their recognition by the recruited phagocytes (44, 45). PS can be detected directly through membrane receptors, such as brain-specific angiogenesis inhibitor 1 (BAI1) (46), stabilin 2 (47, 48), and members of the T cell immunoglobulin mucin domain (TIM) protein family (including TIM1, TIM3, and TIM4) (49–51). The recognition of apoptotic cells can also be mediated indirectly via bridging molecules or accessory receptors, such as MFG-E8, the C-reactive protein, and Gas-6 (52, 53). Engagement of the PS receptors initiates signaling events within the phagocytes that lead to activation of the small GTPase Rac, and subsequent cytoskeletal reorganization, which ultimately leads to engulfment of the apoptotic cell (54, 55).

The engulfment process is not only silent, but also actively anti-inflammatory. Firstly, phagocytes act as “garbage collectors,” which sequester dying cells thus preventing the release of potentially dangerous or immunogenic intracellular contents. Secondly, engulfed phagocytes actively secrete anti-inflammatory cytokines to facilitate the “immunologically silent” clearance of apoptotic cells. These include TGF-β and interleukin (IL)-10, which is even potent enough to suppress LPS-induced inflammatory cytokine release (10, 56, 57). A recent report demonstrates that 12/15-lipoxygenase has been involved in maintaining immunologic tolerance (58). The uptake of apoptotic cells by 12/15-lipoxygenase expressing, alternatively activated resident macrophages blocked the uptake of apoptotic cells into freshly recruited inflammatory Ly6^Chi^ monocytes. Moreover, loss of 12/15-lipoxygenase activity resulted in an aberrant phagocytosis of apoptotic cells by inflammatory monocytes, subsequent antigen presentation of apoptotic cell-derived antigens, and a lupus-like autoimmune disease (58).

If apoptotic cells are not removed promptly they will undergo secondary necrosis and display distinctive morphological changes that can be assessed by flow cytometry (59, 60). Insufficient clearance of dying cells may promote the initiation of autoimmunity and chronic inflammation (61, 62). For example, deregulated apoptosis and insufficient removal of apoptotic cells leads to the release of modified chromatin into the circulation and activation of antigen-presenting cells, which play an important role in the pathogenesis of systemic lupus erythematosus (61, 63). Interestingly, recent studies imply that apoptosis is associated with compensatory proliferation of neighboring cells and plays a pivotal role in modulating tumor cell repopulation (64, 65). For example, Huang et al. reported that dying tumor cells produce PGE2 in a caspase 3-dependent manner and that this has a potent growth-stimulating effect that may stimulate tumor repopulation after radiotherapy (66). The role of further “find me” signals and damage-associated molecular pattern molecules (DAMPs) released by tumor cells killed by chemo- or radiotherapy in the repopulation of the tumor remains elusive. Here, we present a current review that nucleotides derived from dead and dying cells as powerful mediators with broad effects on survival of tumor cells and on the immune system.

## Nucleotides Acting as “Find Me” Signals

It is well established that apoptotic cells release “find me” signals to attract phagocytes and thereby leading to the prompt clearance of the dying cells. The nucleotides ATP and UTP have been recently implicated as a new class of “find me” signals *in vitro* and *in vivo* (39). However, the function of ATP and nucleotides as a find me signal in apoptotic cell clearance is still controversial.

Elliot et al.’s study shows that small amounts of intracellular ATP and UTP are released in a regulated manner during early apoptosis to establish a gradient for monocyte attraction (39). Pannexin 1 channels opening mediate the release of ATP and UTP after caspase-dependent cleavage of their carboxy-terminal tail during apoptosis (67). Several other studies also seem to confirm that nucleotides released from apoptotic cells and subsequent P2Y2 receptor activation promotes monocyte migration by regulating adhesion molecule/chemokine expression in vascular endothelial cells (68, 69). In the neural system, extracellular nucleotides and P2YRs have been implicated in mediating the chemotaxis of microglia toward injured neurons (70, 71).

However, the role of nucleotides in chemotaxis still remains controversial. On the one hand, Elliot et al. could not exclude the possibility that other chemotactic factors participate in the observed chemoattractant effect. On the other hand, nucleotides are unlikely to serve as long-range “find me” signals to phagocytes since they are readily degraded by extracellular nucleotidases (72). Several recent publications do not consider ATP any longer as a “real” direct chemoattractant for macrophages. One study describes ATP as an indirect chemoattractant that steers macrophages in a gradient of the chemoattractant C5a via autocrine release of ATP, generating an amplification of gradient sensing via a “purinergic feedback loop” involving P2Y2 and P2Y12 receptors (73). Hanley et al. confirmed that ATP and ADP leaking from dying cells induce lamellipodial membrane protrusive activity and act as local short-range “touch me” (rather than long-range “find me”) signals to promote phagocytic clearance (74). It is more likely that ATP, together with additional find me signals recruit phagocytes toward injured cells (75, 76). For example, formyl peptides and mitochondrial DNA released from the mitochondria of injured cells have been shown to induce neutrophil activation and chemotaxis in the circulation (77). Formyl peptides, together with chemokines and ATP, synergistically guide and localize phagocytes to sites of sterile inflammation in long-range settings (75). HMGB1 could also synergize with ATP stimulating P2X7 receptors to induce IL-1β release by DCs in contact with dying tumor cells and promoting immunity against tumors (78).

Moreover, nucleotides also play a role in modulating the phagocytic ability or activity of cells surrounding the apoptotic cells. For example, extracellular nucleotides and subsequent P2 receptor (P2X1R, P2X3R) signaling engagement have been reported to enhance the ability of macrophages to bind apoptotic bodies, internalize them and present processed antigens (79). UDP has also been shown to enhance microglia phagocytosis toward apoptotic corpses through the P2Y6 nucleotide receptor during neural inflammation (80).

During tissue injury and/or infection, extracellular nucleotides have been implicated to play a key role in the recruitment of professional phagocytes to sites of tissue injury and/or infection. However, the underlying mechanism is still unclear and not fully understood. It is still debating that extracellular nucleotides act either as chemotactic “find me” signal released by dying cells or through autocrine ATP amplifier signaling for chemotactic navigation to other end-target chemoattractants, such as complement C5a.

## P2 Receptors Signaling in Inflammatory Diseases

**Figure 1:**
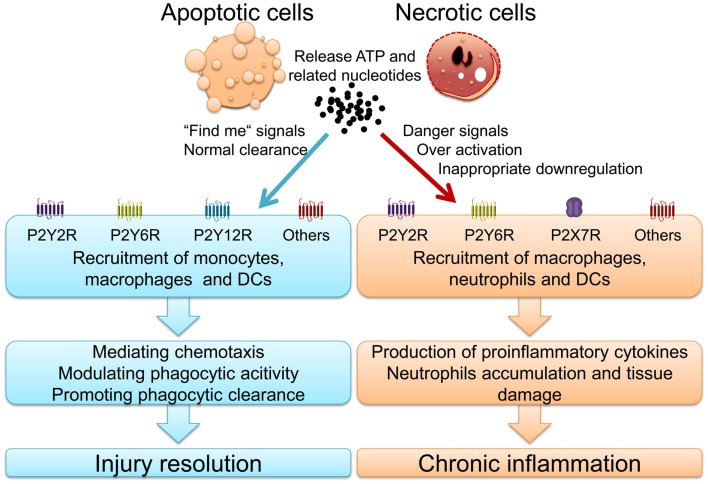
**Nucleotides and activated purinergic signaling during injury resolution and chronic inflammatory diseases**.

Nucleotides release from dying cells and damaged tissues and subsequent purinergic signaling play a pivotal role in phagocytic process and inflammatory diseases (11, 12). For example, P2X7R activation is involved in PS expose in pseudoapoptosis and large amounts of ATP release (81, 82). During the last decade, several studies have highlighted fundamental roles for P2YRs in inflammatory and infectious diseases (Figure 1). Here in particular, signaling events via P2Y2R, P2Y6R, and P2X7R will be discussed thoroughly.

### P2Y2R

P2Y2R has been shown to be up-regulated in a variety of tissues in response to stress or injury and to mediate tissue regeneration through its ability to activate multiple signaling pathways. Many studies implicate that ATP and P2Y2R signaling appears to influence a diverse scale of biological processes such as the generation of chemotactic signals and/or the activation of different immune cells, causing inflammatory cells to migrate, proliferate, differentiate, or release diverse inflammatory mediators (72, 83, 84).

Cystic fibrosis is a life-shortening disease in which airways of the patients are susceptible to infection. Its pathology is characterized by protective and also destructive neutrophilic inflammation. Neutrophil proteases are critical for killing engulfed bacteria, however, neutrophilic elastase accumulation in the airways of patients with cystic fibrosis (CF) overwhelms antiprotease defenses, resulting in impaired ciliary function, crippling bacterial clearance, and degrading structural proteins, eventually leading to bronchiectasis (85). CF results from a variety of mutations in the gene encoding the CF transmembrane conductance regulator (CFTR) protein, a cAMP-regulated chloride channel in epithelial cells, which will lead to sodium hyperabsorption in the airway of patients with CF (86, 87). Mucociliary clearance in CF lung is limited by airway dehydration, leading to persistent bacterial infection and inflammation. P2Y2 receptors have been shown to regulate chloride secretion and sodium absorption on epithelial cells in distal bronchi (88). Moreover, ATP, acting through P2Y2 receptors, regulates the secretion of ions, mucin, and surfactant phospholipids in respiratory epithelium (89). Several studies have shown that P2 receptor purinergic compounds are explored for the treatment of CF, to bypass the defective function of CFTR, and to restore chloride secretion and/or inhibit sodium absorption through inhibiting the epithelial sodium channel ENaC expression (90). P2Y2R agonists increase the duration of mucociliary clearance stimulation. The efficacy and safety of the P2Y2R agonist denufosol has been evaluated in several clinical trials, however, long term follow-up results do not show any improvement in pulmonary function (91, 92).

P2Y2R is not only involved in enhancing mucociliary clearance, but also plays a role in promoting wound healing (93). Damaged fibroblasts release ATP or UTP and activate P2Y2R to enhance the proliferation and migration of fibroblasts. Wound size in WT mice decreases significantly compared to P2Y2R^−/−^ mice, and WT mice express proliferation marker Ki67 and extracellular matrix (ECM)-related proteins VEGF. It indicates that triggering of P2Y2R may be a potential therapeutic target to promote wound healing (94).

Adenosine 5′-triphosphate has also been implicated to induce chemotaxis of neutrophils via actin polymerization and direct cell orientation by feedback signaling involving P2Y2R (95–97). The subsequent P2Y2R activation will amplify gradient sensing of chemotactic signals (e.g., N-formyl peptides and IL-8) by stimulating F-actin to the leading edge (97–99). Chemotaxis of neutrophils to sites of infection is critical for immune defense and for the physiological downregulation of neutrophil-driven inflammation (100).

However, excessive accumulation of neutrophils through inappropriate activation of P2Y2R can cause acute tissue damage during sepsis, chronic obstructive pulmonary disease (COPD), and hepatitis (101–104). COPD is one of the most common inflammatory diseases and is associated with inflammation of the small airways, which results in airway obstruction, destruction of parenchyma, and development of emphysema (105). ATP and activation of P2Y2R contribute to smoke-induced lung inflammation and to the subsequent development of emphysema (104). ATP acts as a “danger signal” recruiting neutrophils to the lung and inducing inflammation. P2Y2R^−/−^ mice show reduced pulmonary inflammation and less emphysema development after short-term smoke exposure. ATP enhances chemotaxis and elastase release in blood neutrophils from patients with COPD, compared to normal healthy subjects (103).

In asthmatic chronic airway inflammation, P2Y2R has been indicated as a critical sensor for airway exposure to airborne allergens by mediating ATP-triggered migration of immature monocyte-derived DCs and eosinophils in both, mice and humans (106, 107). This process is accompanied with the production of pro-allergic mediators (for example, IL-33, IL-8, eosinophil cationic protein) from different cellular sources (107, 108). Moreover, heightened expression and localization patterns of P2YR are associated with chronic pancreatic diseases (109).

In summary, ATP and P2Y2R signaling is a double-edged sword. On the one hand, it can protect against infections, promote wound healing and enhance mucociliary clearance. On the other hand, it can also lead to uncontrolled inflammation and promotion of chronic inflammatory disease states and fibrotic remodeling (Figure 1) (109). Indeed, P2Y2R may be a new target for therapy of COPD and P2Y2R antagonists could be useful drugs for chronic inflammatory diseases.

### P2Y6R

Similar to P2Y2R, P2Y6R plays an ambivalent role in inflammatory diseases. The receptor is crucial for innate immune responses against bacterial infection (110). Many studies show that P2Y6R activation is involved in the release of chemokines from immune cells, such as monocytes, DCs, eosinophils, and recruiting monocytes/macrophages during inflammation or infection (24, 110–114).

In neurodegenerative diseases, microglia are engaged in the clearance of dead cells or dangerous debris, which is crucial for the maintenance of brain functions. Extracellular ATP regulates microglial motility dynamics in the intact brain, and its release from the damaged tissues mediates a rapid microglial response toward injury (71). Moreover, UTP and UDP released from injured neurons have been shown to enhance microglial phagocytic capacity for dying cells via activation of P2Y6R, serving as an “eat-me” signal for microglia. This signal is considered to be an important initiator of the clearance of dying cells or debris in the CNS (80).

However, P2Y6R signaling is relatively harmful in endothelial or epithelial inflammation (111, 115). The idiopathic inflammatory bowel diseases (IBD) comprise two types of chronic intestinal disorders: Crohn’s disease and ulcerative colitis, which result from an inappropriate inflammatory response to intestinal microbes in a genetically susceptible host (116). Up-regulation of P2Y2R and P2Y6R in intestinal epithelial cells has been reported in experimental colitis (115).

Similarly, P2Y6R plays an important role in acute and chronic allergic airway inflammation, and selective blocking of P2Y6R or P2Y6R deficiency in structural cells reduces symptoms of experimental asthma. Recently, P2Y6 receptors have not only been found to be up-regulated in murine atherosclerotic plaques, but also to play a key role in MSU-associated inflammatory diseases (117, 118).

Thus, P2Y6R activation plays a role in innate immunity against infection whereas P2Y6R over-activation can result in harmful immune responses and chronic inflammation such as atherosclerosis, COPD, and IBD (Figure 1).

### P2X7R

P2X7R are predominantly expressed on immune cells such as mast cells, macrophages, microglia, and dendritic cells (119). Many evidences implicate the role of P2X7R against microbes during inflammation and immune response (120, 121). Indeed, P2X7R signaling plays a key role in immune responses against bacterial and parasitic infection. It has been reported that P2X7R signaling is involved in the elimination of intracellular microbes – such as *Mycobacterium tuberculosis*, *Chlamydia trachomatis*, and *Leishmania amazonensis* – either by contributing to killing of the pathogen or by inducing cell death of infected macrophages (121). P2X7R is also involved in fever development via PGE2 and IL-1β production (122).

The P2X7R is widely recognized to mediate the pro-inflammatory effects of extracellular ATP. However, recently one study revealed that P2X7 receptor also acts as one of the scavenger receptor involved in the recognition and removal of apoptotic cells in the absence of extracellular ATP and serum (123). The P2X7R has drawn particular attention as a potential drug target due to its broad involvement in inflammatory diseases (124).

In the CNS, P2X7R activation contributes to neuroinflammation through the release of pro-inflammatory cytokines, such as IL-1β and TNF-α (125, 126). It also activates MAP kinases and NF-κB, resulting in up-regulation of pro-inflammatory gene products, including COX-2 (127) and the P2Y2R (128). Alzheimer’s disease (AD) is the most common form of dementia and more than 35 million people worldwide suffer from AD (129). The appearance of plaques consisting of extracellular β-amyloid peptide (Aβ) is a neuro-pathological feature of AD, which is surrounded by reactive microglial cells (129, 130). In P2X7R^-/-^ mice, Aβ triggered increase of intracellular Ca^2+^, ATP release, IL-1β secretion, and plasma membrane permeabilization in microglia (131). In fact, *in vivo* inhibition of P2X7R in mice transgenic for mutant human amyloid precursor protein (APP) indicated a significant decrease of the number of hippocampal amyloid plaques (132). Thus, the identification of extracellular ATP and P2X7R as key factors in Aβ-dependent microglia activation unveils a non-conventional mechanism in neuroinflammation and suggests new possible pharmacological targets.

Extracellular ATP and P2X7R signaling also contributes to the development of smoking-induced lung inflammation and emphysema. P2X7R^-/-^ mice exhibit decreased inflammatory responses, including a reduction in pulmonary fibrosis in a mouse model of lung inflammation (133). Inhibition of this receptor may be a new possible therapeutic target for the treatment of COPD (133, 134).

The purinergic P2X7R is associated with activation and release of IL-1 and IL-18, which is strongly implicated in the multiple inflammatory pathways involved in the pathogenesis of rheumatoid arthritis (RA) (135–139). P2X7R has also been shown to be expressed by synoviocytes from RA joints and contributes to modulation of IL-6 release (140). P2X7R activation also plays a novel and direct role in tissue damage through release of cathepsins in joint diseases (141). Although, AZD9056, a P2X7R antagonist, has been shown to reduce articular inflammation and erosive progression (142), clinical trials with the P2X7R antagonist in patients with RA failed to inhibit disease progression (143, 144). Similarly, the effect and safety of AZD9056 in Crohn’s disease is still under clinical trial (145).

Taken together, P2X7R signaling not only plays a critical role in mediating appropriate inflammatory and immunological responses against invading pathogens, but also contributes to a wide range of chronic inflammatory diseases when activated inappropriately (Figure 1).

## Conclusion

The interaction between dying cells and phagocytes is very complex and nucleotides have been involved in orchestrating the process of dead cell removal. On the one hand, nucleotides and purinergic signaling have been shown to play a key role in the apoptotic cell clearance avoiding secondary necrosis, preventing inflammation and contributing to regeneration of injured tissues. On the other hand, purinergic signaling over-activation is involved in chronic inflammation and chronic inflammatory diseases. Adenosine-mediated P1 and nucleotides-mediated P2 signaling frequently have opposing effects in biological systems, and shifting the balance between P1 and P2 signaling is an important therapeutic concept in efforts to dampen pathological inflammation and promote healing (12). Nucleotides and purinergic signaling might be used as biomarkers for various diseases and could also provide potential novel therapeutic targets for the treatment of chronic inflammatory diseases.

## Conflict of Interest Statement

The authors declare that the research was conducted in the absence of any commercial or financial relationships that could be construed as a potential conflict of interest.
